# Phenotype, genotype, and worldwide genetic penetrance of *LRRK2*-associated Parkinson's disease: a case-control study

**DOI:** 10.1016/S1474-4422(08)70117-0

**Published:** 2008-07

**Authors:** Daniel G Healy, Mario Falchi, Sean S O'Sullivan, Vincenzo Bonifati, Alexandra Durr, Susan Bressman, Alexis Brice, Jan Aasly, Cyrus P Zabetian, Stefano Goldwurm, Joaquim J Ferreira, Eduardo Tolosa, Denise M Kay, Christine Klein, David R Williams, Connie Marras, Anthony E Lang, Zbigniew K Wszolek, Jose Berciano, Anthony HV Schapira, Timothy Lynch, Kailash P Bhatia, Thomas Gasser, Andrew J Lees, Nicholas W Wood, on behalf of the International LRRK2 Consortium

**Affiliations:** a Department of Clinical Neurosciences, Institute of Neurology, University College London, London, UK; b Genomic Medicine, Imperial College, London and Twin Research and Genetic Epidemiology Unit, St. Thomas Campus, Kings College London, UK; c Reta Lila Weston Institute for Neurological Studies, University of London, UK; d Department of Clinical Genetics, Erasmus MC, Rotterdam, Netherlands; e Institut National de la Santé et de la Recherche Médicale U679, Neurologie et Thérapeutique Expérimentale, Hôpital de la Pitié-Salpêtrière, Assistance Publique-Hôpitaux de Paris, Paris; f Fédération de Neurologie, Centre Hospitalier Universitaire Pitié-Salpêtrière, Assistance Publique-Hôpitaux de Paris, Paris; g Département de Génétique, Cytogénétique et Embryologie, Hôpital de la Pitié-Salpêtrière, Assistance Publique-Hôpitaux de Paris, Paris; h Beth Israel Medical Centre, Department of Neurology, NY and the Albert Einstein College of Medicine, Department of Neurology, Bronx, NY, USA; i Department of Neurology, St Olav's Hospital and Department of Neuroscience, NTNU, 7006 Trondheim, Norway; j Geriatric Research Education and Clinical Centre, Veterans Affairs Puget Sound Health Care System, and Department of Neurology, University of Washington, Seattle, WA, USA; k Parkinson Institute, Istituti Clinici di Perfezionamento, Milan, Italy; l Neurological Clinic Research Unit, Institute of Molecular Medicine, Lisbon School of Medicine, Portugal; m Neurology Service, Institut Clinic Maltias del Sistema Nervios, Hospital Clinic Universitari, University of Barcelona, Spain; n Division of Genetic Disorders, Wadsworth Centre, New York State Department of Health, Albany, NY, USA; o Department of Neurology, University of Luebeck, Luebeck, Germany; p Faculty of Medicine (Neurosciences), Monash University, Melbourne, Australia; q University of Toronto, Toronto, Canada; r Department of Neurology, Mayo Clinic Jacksonville, Florida, USA; s Service of Neurology, University Hospital “Marques de Valdecilla”, (CIBERNED), Santander, Spain; t Department of Neurology, Mater Misericordiae University Hospital, and the Conway Institute of Biomolecular and Biomedical Research, Dublin, Ireland; u Sobell Department of Motor Neuroscience and Movement Disorders, Institute of Neurology, Queen Square, London, UK; v Department of Molecular Pathogenesis, Institute of Neurology, Queen Square, London, UK; w Department of Neurodegenerative Diseases, Hertie-Institut for Clinical Brain Research, University of Tuebingen, Germany

## Abstract

**Background:**

Mutations in *LRRK2*, the gene that encodes leucine-rich repeat kinase 2, are a cause of Parkinson's disease (PD). The International *LRRK2* Consortium was established to answer three key clinical questions: can *LRRK2*-associated PD be distinguished from idiopathic PD; which mutations in *LRRK2* are pathogenic; and what is the age-specific cumulative risk of PD for individuals who inherit or are at risk of inheriting a deleterious mutation in *LRRK2*?

**Methods:**

Researchers from 21 centres across the world collaborated on this study. The frequency of the common LRRK2 Gly2019Ser mutation was estimated on the basis of data from 24 populations worldwide, and the penetrance of the mutation was defined in 1045 people with mutations in *LRRK2* from 133 families. The *LRRK2* phenotype was defined on the basis of 59 motor and non-motor symptoms in 356 patients with *LRRK2*-associated PD and compared with the symptoms of 543 patients with pathologically proven idiopathic PD.

**Findings:**

Six mutations met the consortium's criteria for being proven pathogenic. The frequency of the common LRRK2 Gly2019Ser mutation was 1% of patients with sporadic PD and 4% of patients with hereditary PD; the frequency was highest in the middle east and higher in southern Europe than in northern Europe. The risk of PD for a person who inherits the LRRK2 Gly2019Ser mutation was 28% at age 59 years, 51% at 69 years, and 74% at 79 years. The motor symptoms (eg, disease severity, rate of progression, occurrence of falls, and dyskinesia) and non-motor symptoms (eg, cognition and olfaction) of *LRRK2*-associated PD were more benign than those of idiopathic PD.

**Interpretation:**

Mutations in *LRRK2* are a clinically relevant cause of PD that merit testing in patients with hereditary PD and in subgroups of patients with PD. However, this knowledge should be applied with caution in the diagnosis and counselling of patients.

**Funding:**

UK Medical Research Council; UK Parkinson's Disease Society; UK Brain Research Trust; Internationaal Parkinson Fonds; Volkswagen Foundation; National Institutes of Health: National Institute of Neurological Disorders and Stroke and National Institute of Aging; Udall Parkinson's Disease Centre of Excellence; Pacific Alzheimer Research Foundation Centre; Italian Telethon Foundation; Fondazione Grigioni per il Morbo di Parkinson; Michael J Fox Foundation for Parkinson's Research; Safra Global Genetics Consortium; US Department of Veterans Affairs; French Agence Nationale de la Recherche.

## Introduction

Parkinson's disease (PD) affects 1–2% of people older than 65 years. Respective mutations in five genes—*SNCA* (α-synuclein), *PARK2* (parkin), *PARK7* (DJ-1), *PINK1*, and *LRRK2*—can cause parkinsonism that resembles idiopathic PD.^1^ *LRRK2* encodes the 51-exon, multidomain protein, leucine-rich repeat kinase 2 (LRRK2); mutations in *LRRK2* cause an autosomal dominant PD that, in most patients, produces a α-synuclein-type neuropathology.^2^, ^3^, ^4^

Although individual *LRRK2* genotype–phenotype correlations have been reported,^5^, ^6^, ^7^ these have been reported for a mean of only five mutation carriers per study, and the results can not be easily compared because of the use of different clinical approaches, sample sizes, diagnostic criteria, and genotyping techniques.

The International *LRRK2* Consortium was established to pool worldwide data to answer important clinical questions through collective experience and research. Three questions have been considered by the consortium: can *LRRK2*-associated PD be distinguished from idiopathic PD; which mutations in *LRRK2* are pathogenic; and what is the age-specific cumulative risk of PD for individuals who inherit or are at risk of inheriting a deleterious mutation in *LRRK2*?

## Methods

### Patients and procedures

Researchers at 21 primary centres collaborated on this study. The clinical and genetic data were collected and analysed at The National Hospital for Neurology and Neurosurgery, Queen Square, London, UK. The study was approved by the joint Research Ethics Committee of the Institute of Neurology and the National Hospital for Neurology and Neurosurgery, London, UK. Informed consent was obtained from each patient by the local ethics committees.

Clinical data were collected at each participating centre on a standardised, two-part pro forma questionnaire. Each patient was assessed by a neurologist who was a specialist in movement disorders, and a separate pro forma was completed for each patient. The average time to complete the pro forma was 21 mins per patient. Most of the clinical data were collected prospectively and not specifically for this project. The first part of the pro forma consisted of 13 sections that focused on population demographics and genotyping techniques for the patients and controls. Each centre provided details on the number of patients with PD who were screened for mutations in *LRRK2*, their ethnic backgrounds, and the number of controls used, to confirm the pathogenicity of novel mutations. The second part of the pro forma was a 59-section clinical questionnaire on motor and non-motor symptoms. The pro formas were completed by the neurologist.

Validated scales were used to assess olfaction (University of Pennsylvania smell identification test [UPSIT]),^8^ cognition (the mini-mental state examination),^9^ and to stage the disease and its progression (the Hoehn and Yahr scale^10^). We attempted to assess the severity of other symptoms, such as anxiety, depression, sleep disturbance, dysautonomia, and dystonia, with unified standardised criteria; however, this was difficult to standardise across centres, and instead we chose a simplified “symptom present”, “symptom absent”, or “symptom not recorded” scoring method because it was more reproducible.

The rate of disease progression was measured by the time taken to reach each stage on the Hoehn and Yahr scale and the percentage of patients at each stage of the scale with PD for longer than 10 years, starting from symptom onset rather than time of diagnosis.

The clinical features of mutations in *LRRK2*-associated PD were compared with a series of 543 clinically documented, pathologically proven cases of idiopathic PD from the Queen Square Brain Bank (QSBB). All QSBB patients were screened for the LRRK2 Gly2019Ser mutation, and carriers were excluded. The same phenotyping methods were used for patients with mutations in *LRRK2* as were used in the QSBB group.

Mutation-screening methods were not standardised; however, most centres used direct DNA sequencing, and we relied on the quality controls at each participating centre. Mutations were arbitrarily designated as ‘proven’ pathogenic if they were found in three or more unrelated participants, or if the mutation segregated within a large family with many members who had PD and was not found in more than 1000 ethnically matched controls. The consortium acknowledges that many reported mutations do not fulfil these criteria but are likely to be pathogenic.

### Statistical analysis

The penetrance of all mutations in *LRRK2* (age-specific cumulative risk of PD) was estimated with the Kaplan-Meier life table survival method and maximum-likelihood estimates calculated with the pedigree analysis package for Java (jPAP Version 1.5.0).^11^, ^12^ Kaplan-Meier survival function was calculated for mutation carriers and individuals whose probability of carrying a mutation was greater than 90% (as inferred from jPAP) in affected and unaffected individuals.^13^ No minimum size was imposed on families (ie, the analysis was not restricted to large families with many affected [penetrating] members). To further minimise any ascertainment bias, the index case of each pedigree was excluded. Similarly, sporadic (singleton) cases were excluded when data were not available on at least both parents. A Kaplan-Meier curve to estimate the time to onset of PD was plotted using the R project for statistical computing,^14^ and the curve was inverted to give an empirical, cumulative-risk step function.

Maximum-likelihood estimates of age-specific penetrance were calculated by maximising the log likelihood of seeing the variant genotypes while restricting affection probabilities to age-specific incidence intervals. PD incidence rates were based on previous estimates in 588 patients.^15^ The frequency of the disease allele was set to 0·001. The model assumed that an earlier disease onset corresponded to a higher liability. The incidences determine a series of points that are ordered inversely on the liability scale. The genotype-specific probability of an individual developing PD at a particular age was evaluated within the corresponding liability interval. For an unaffected individual, the age was right censored at the age of last follow-up or death, and the genotype-specific affection probability evaluated from the upper limit of that liability interval to infinity. The sample had ascertainment correction as per the method of Cannings and Thompson.^16^

The variant effect was characterised by dominance and displacement. If the disease allele is denoted as A, the dominance is calculated by the rate of differences between the mean probabilities of the heterozygous and homozygous carriers of the disease-variant ([μAa–μaa]/[μAA–μaa]) and the wild-type homozygotes. The dominance was set to equal 1 in this analysis, to investigate the variant effect with a dominant contribution towards the phenotype. The displacement is the difference between the mean liabilities of the homozygotes relative to the total standard deviation within genotypes (μ_AA_–μ_aa_)/σ. The total mean liability and the variance (σ^2^) were restricted to equal 0 and 1, respectively. Ascertainment correction was done by maximising the conditional likelihood of the pedigree, given the phenotypic and genotypic information of the index case. A likelihood-ratio test was used to assess the heterogeneity of risk by sex, ethnic group (white or not white), and mutation. Owing to the high frequency of LRRK2 Gly2019Ser carriers in our sample, all the other pathogenic mutations were analysed together.

### Role of the funding source

The study sponsors had no role in the study design, the conduct of the study, data collection, data analysis, data interpretation, nor writing of the report. The investigators had full responsibility for submitting the manuscript for publication. The corresponding author had full access to all of the data in the study and takes responsibility for the integrity of the data and the accuracy of the analysis.

## Results

19 376 unrelated patients with PD were genotyped for *LRRK2* mutations. *LRRK2* 6055G→A, the mutation that causes LRRK2 Gly2019Ser, was found in 201 families and in 179 patients with apparently sporadic PD. Table 1 summarises the frequency of LRRK2 Gly2019Ser in 24 populations; there were insufficient data to estimate the population frequencies of mutations other than LRRK2 Gly2019Ser; therefore, clinical data are reported as all mutations in *LRRK2*, the combination of mutations in *LRRK2* other than *LRRK2* 6055G→A, and *LRRK2* 6055G→A (LRRK2 Gly2019Ser) only.

**Table 1:** Frequency of LRRK2 Gly2019Ser in patients with PD and controls across 24 world populations

	**Patients with sporadic PD**		**Patients with hereditary PD**		**Controls**	
	N	Patients with mutations (%)	N	Patients with mutations (%)	N	Patients with mutations (%)
North African Arabs	56	22 (39%)	143	51 (36%)	739	4 (<1%)
Ashkenazi Jews	259	25 (10%)	78	22 (28%)	410	4 (1%)
Portuguese	317	13 (4%)	85	12 (14%)	100	0 (0%)
Chilean*	137	4 (3%)	29	1 (3%)	153	0 (0%)
Spanish	806	22 (3%)	283	14 (4%)	544	0 (0%)
Swedish*	200	4 (2%)	127	0 (0%)	200	0 (0%)
French	300	5 (2%)	174	5 (3%)	348	0 (0%)
Italian and Sardinian	2516	37 (2%)	633	26 (4%)	1040	1 (<1%)
North American (white)	2606	26 (1%)	1450	45 (3%)	4934	1 (<1%)
British	1145	9 (1%)	192	4 (2%)	1786	0 (0%)
Norwegian	371	3 (1%)	64	6 (1%)	572	0 (0%)
Russian*	157	1 (1%)	10	0 (0%)	126	0 (0%)
Irish	236	1 (<1%)	35	1 (3%)	212	0 (0%)
Greek*	235	1 (<1%)	0	0 (0%)	0	0 (0%)
German and Austrian	803	2 (<1%)	231	3 (1%)	436	0 (0%)
Australian*	578	2 (<1%)	252	6 (2%)	0	0 (0%)
Japanese*	526	1 (<1%)	60	1 (2%)	372	1 (<1%)
Indian*	718	1 (<1%)	82	0 (0%)	1200	0 (0%)
Serbian*	47	0 (0%)	51	2 (4%)	161	0 (0%)
Cretan	174	0 (0%)	92	1 (1%)	0	0 (0%)
Chinese*	1360	0 (0%)	973	1 (<1%)	938	0 (0%)
Basque	117	0 (0%)	41	0 (0%)	425	0 (0%)
Korean*	436	0 (0%)	17	0 (0%)	0	0 (0%)
Polish*	153	0 (0%)	21	0 (0%)	190	0 (0%)
Total white	10714	126 (1%)	3690	123 (3%)	10913	2 (<1%)
Total worldwide	14253	179 (1%)	5123	201 (4%)	14886	11 (<1%)

PD was subdivided into those with an affected first-degree relative (hereditary) and those without a family history of PD (sporadic/singleton).

* No clinical data were received from these populations; estimates are based on published data.

The worldwide frequency of LRRK2 Gly2019Ser was 1% of patients with sporadic PD and 4% of patients with hereditary PD (table 1). The highest frequency of LRRK2 Gly2019Ser was seen in north African Arabs (hereditary 36%, sporadic 39%) and Ashkenazi Jews (hereditary 28%, sporadic 10%). The frequency was higher in southern European countries than in northern European countries. LRRK2 Gly2019Ser was rarely seen in Asians (Chinese, Japanese, Korean, and Indian); it was found in only four of 4172 patients (<0·1%). Completed pro formas were available for 356 patients with PD (hereditary or sporadic) who had any mutation in *LRRK2*, 313 of whom had LRRK2 Gly2019Ser. The mean disease duration was shorter for patients with any mutation in *LRRK2* than for patients with idiopathic PD (10·9 years [SD 7·8 years] *vs* 15·1 years [SD 7·1 years], mean difference 4·2 years, 95% CI 3·2–5·2; p<0·0001).

The mean age of PD onset for all *LRRK2* mutation carriers was 58·1 years (14·0 years). This did not differ significantly by sex, and was similar in patients with LRRK2 Gly2019Ser (57·5 years [13·9 years] and carriers of all LRRK2 non-Gly2019Ser mutations (59·9 years [13·8 years]). Patients in the QSBB series developed PD at a slightly older age than *LRRK2* mutation carriers (61·0 years [10·9 years], mean difference 2·9 years, 95% CI 1·3–4·5; p<0·0001). 15 patients (3%) with LRRK2 Gly2019Ser developed PD after the age of 80 years, including five patients who developed PD after 90 years.

**Figure 1:**
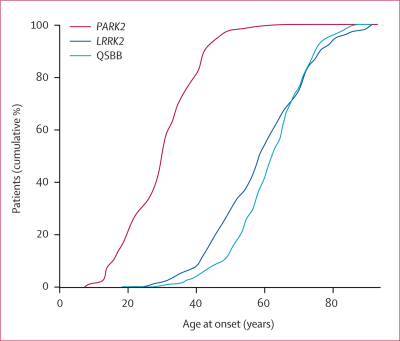
Age of PD onset plotted against the cumulative percentage of patients in the *PARK2, LRRK2*, or QSBB series QSBB=Queen Square brain bank.

Mutations in *PARK2* (parkin) are the second most common genetic cause of parkinsonism after mutations in *LRRK2*.^17^, ^18^ Across the consortium, we identified 184 homozygous carriers of *PARK2* mutations who had a mean age of PD onset of 29·2 years (10·4 years). Figure 1 shows the age of onset versus the cumulative percent of patients with mutations in *LRRK2, PARK2*, or in the QSBB series. *LRRK2*-associated PD develops at a younger age than the age of onset in the QSBB group; however, although the onset of *LRRK2*-associated PD occurs at a slightly younger age than idiopathic PD does, it is of little clinical relevance. More clinically useful is the difference in the age of PD onset between the patients with *LRRK2* mutations and those in the QSBB series, and patients who are homozygous for mutations in *PARK2*. Only 29 *LRRK2* mutation carriers (8%) or 25 patients with idiopathic PD (4%) developed symptoms of PD before the age of 40 years; by contrast, 155 (84%) of the patients who were homozygous for *PARK2* mutations had presented with symptoms of PD by the age of 40 years.

The patients with mutations in *LRRK2* had a mean duration from the onset of PD to Hoehn and Yahr scale stage 2 (bilateral symptoms, no difficulty walking) of 7·2 years (SD 4·1 years) and a similar duration was seen between LRRK2 Gly2019Ser and LRRK2 non-Gly2019Ser carriers; however, the numbers in the latter group were small and did not include many different mutations. There was no difference when adjusted for age or sex. The rate of disease progression in *LRRK2* mutation carriers measured by the time to progression through each point on the Hoehn and Yahr scale and the percentage of those patients at each stage of the Hoehn and Yahr scale that had symptoms of PD for more than 10 years are summarised in table 2.

**Table 2:** Rate of disease progression in patients with all *LRRK2* mutations and LRRK2 Gly2019Ser

	**Time of progression to H and Y stage**		**Proportion of patients at each H and Y stage with PD for 10 years or longer**	
	All mutations (n=321)	Gly2019Ser (n=291)	All mutations (n= 257)	Gly2019Ser (n=226)
H and Y stage 1	4·0 years	4·1 years	6%	6%
H and Y stage 2	7·2 years	7·4 years	27%	31%
H and Y stage 3	9·4 years	9·1 years	45%	44%
H and Y stage 4	12·6 years	12·9 years	57%	61%
H and Y stage 5	15·6 years	16·2 years	79%	82%

Average number of years it took each patient to reach each stage of the H and Y scale and the percentage of patients at each stage who had symptoms for 10 years or longer. H and Y=Hoehn and Yahr scale

For clinicians, the occurrence of falls is a more practical measure of the severity of disease than disease rating scales. The mean time to first fall in patients with a mutation in *LRRK2* was longer than in the QSBB series (12·6 years [SD 7·9 years] *vs* 9·3 years [SD 5·9 years]; difference 3·3 years, 95% CI 2 ·4–4·2 years; p<0·0001).

At some point during the course of the disease, tremor, bradykinesia, and rigidity were seen in 93% (331) of patients with mutations in *LRRK2* and 94% (510) of patients in the QSBB series. Tremor was the most common presenting symptom in both groups (63% [224] of patients with mutations in *LRRK2* and 52% [282] in the QSBB series), then bradykinesia (27% [92] and 36% [198], respectively), and rigidity (10% [36] and 12% [65], respectively). The higher incidence of tremor in the patients with mutations in *LRRK2* than the QSBB was significant (odds ratio [OR] 1·49, 95% CI 1·1–2·0; p<0·003). Of the 271 patients (76%) with mutations in *LRRK2* who had descriptive accounts of their tremor, the tremors were characterised as ‘rest’ tremor in 73%, and ‘leg’ tremor—described by four independent centres as an abduction–adduction leg movement—was a first symptom in 9% of patients, compared with only three of 193 patients (2%) in the QSBB series who had descriptive accounts of their tremor.

Any form of dystonia was seen in 126 of 301 patients (42%) with mutations in *LRRK2* compared with 121 of 487 patients (25%) with idiopathic PD; in most patients this was a painful “off period” foot dystonia. Dystonia occurred during the first 2 years of the disease in 22 of 126 patients (18%) with mutations in *LRRK2* compared with 5 of 21 (4%) of patients with idiopathic PD (OR 4·5, 95% CI 2·4–8·4; p<0·0001). Only one patient with a mutation in *LRRK2* had dystonia before dopamine-replacement treatment. Atypical examples of dystonia that affected the arm (n=2), neck (n=2), tongue (n=1), and that caused blepharospasm (n=4) were also reported in patients with mutations in *LRRK2*.

Dopamine-replacement regimens varied across centres, but the clinical assessment of the responses were good or excellent in 88% (313 of 356) of patients, modest in 9% (32), and poor in 3% (10). The clinical responses were similar to those in patients with idiopathic PD (good or excellent in 83% [450 of 543], modest in 12% [65], and poor in 5% [28]). Patients with idiopathic PD needed treatment earlier than patients with mutations in *LRRK2*: the mean time from the onset of PD to the start of dopamine-replacement treatment was 4·01 years (SD 2·50 years) for patients with mutations in *LRRK2* and 3·03 years (2·90 years) for patients with idiopathic PD (difference 0·98 years, 95% CI 0·61–1·35 years; p<0·0001). 66 (19%) patients with mutations in *LRRK2* compared with 38 (7%) patients with idiopathic PD (p=0·003) were not on dopamine-replacement treatment 5 years after disease onset.

Drug-induced, interdose dyskinesia was reported by 206 (58%) patients with mutations in *LRRK2* and by 293 (54%) patients in the QSBB group. Although the incidence of dyskinesia was similar in both groups, the time to onset was longer in patients with mutations in *LRRK2* than in patients with idiopathic PD (8·4 years [SD 4·6 years] *vs* 5·6 years [SD 3·7 years], difference 2·8 years, 95% CI 2·3–3·3 years; p<0·0001). Only 39 (11%) patients with mutations in *LRRK2* were dyskinetic after 5 years of treatment, and only 114 (32%) were dyskinetic after 10 years of treatment. By comparison, 136 (25%) patients with idiopathic PD were dyskinetic after 5 years of treatment and 223 (41%) patients were dyskinetic after 10 years of treatment.

Stereotactic functional neurosurgery was done on 22 patients with mutations in *LRRK2*: 18 had unilateral or bilateral subthalamic nucleus stimulation, three had pallidotomy, and one had thalamotomy. The mean time from PD onset to surgery was 11·4 years (SD 6·2), and the indications were usually either motor fluctuation or dyskinesia. Of the 12 patients who had detailed measurements of clinical outcome, eight were good or excellent, two moderate, and two poor.

**Figure 2:**
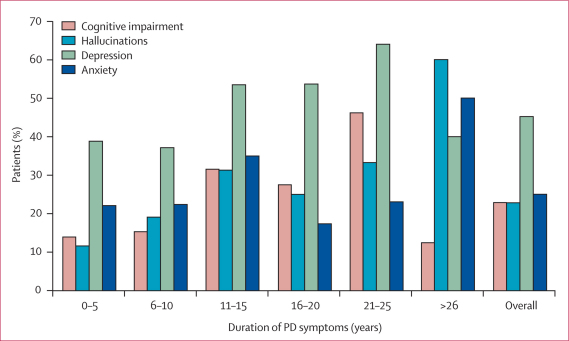
Point prevalence of neuropsychiatric symptoms in patients with mutations in *LRRK2* after various durations of the symptoms of PD

The sample sizes for each non-motor symptom were reduced owing to missing data or not using validated or self-reported diagnostic scales in routine practice. Formal mini-mental state examination data on cognition were obtained on 162 patients with mutations in *LRRK2*. These results and those for other neuropsychiatric symptoms are shown in figure 2. 37 patients (23%) with mutations in *LRRK2* had evidence of cognitive impairment (mini-mental state examination score ≤24) compared with 340 patients (70%) with idiopathic PD. The mean disease duration of patients with cognitive impairment was 15·2 years (SD 5·9 years) for those with mutations in *LRRK2* and 14·4 years (SD 5·9 years) for those with idiopathic PD. However, this must be considered in light of a mean 4·2 years (3·2–5·2 years) longer duration for the entire QSBB series. A more comparable measure is the proportion of patients who develop cognitive impairment within 2 years of symptom onset: 6 patients (3·4%) with mutations in *LRRK2* compared with 48 patients (9·8%) with idiopathic PD (p=0·0016).

The response rate for olfaction was low (n=43) because only UPSIT data were accepted. Abnormal olfaction was found in 22 patients (51%) with LRRK2 Gly2019Ser after a mean disease duration of 5·6 years (SD 4·3).

Urinary symptoms affected 58 of 204 (28%) of the patients with LRRK2 Gly2019Ser, but these did not vary significantly when stratified by disease duration or by mutation. The most common symptoms were frequency and urge incontinence. 48% (93 of 194) of patients had constipation. 11% of men with LRRK2 Gly2019Ser reported erectile dysfunction, all of whom were older than 60 years.

69% (186 of 268) of patients with LRRK2 Gly2019Ser had sleep disturbances; however, there was no significant difference from the controls when this symptom was stratified by disease duration or by mutation. Insomnia and sleep fragmentation were the most common symptoms. Formal sleep studies identified 13 patients with rapid eye movement (REM) sleep behaviour disorder and five patients with restless legs syndrome. However we do not have accurate frequency estimates for these two symptoms because most patients did not have formal sleep studies.

9% (30 of 342) of patients with *LRRK2*-associated PD had diabetes mellitus, which was unexpectedly high and might be due to the effect of multiple-comparison testing. The mean age of these diabetic patients was 54 years (range 42–66 years) but we could not establish the age of diabetes onset. The prevalences of other common medical conditions were similar to those reported for the general population.

There were no apparent differences between the patients with mutations in *LRRK2* and those with idiopathic PD groups with respect to employment history, exposure to toxins, or educational status; however, with the exception of smoking history (n=306), these sections of the pro forma were poorly completed. Smoking (past or present) was seen in 28% of patients with LRRK2 Gly2019Ser compared with 39% of the QSBB series. There was no correlation between smoking history and age of symptom onset.

In this study, six mutations in *LRRK2* met the consortium's criteria for being ‘proven pathogenic’: Gly2019Ser (n=391), Arg1441Gly (n=33), Arg1441Cys (n=9), Arg1441His (n=5), Ile2020Thr (n=5), and Tyr1699Cys (n=2), where n is the number of unrelated individuals or families that carry the mutation. Conclusions made about mutations of low frequency are likely to be less robust than those drawn from mutations of higher frequency.

Penetrance estimates were calculated for LRRK2 Gly2019Ser on the basis of results from 1045 patients in 133 families. These comprised 327 affected patients (index and non-index cases) and 718 unaffected participants, with a mean of 8 individuals per family (range 3–45). Sporadic (singleton) cases were included when there were data on age and affection status from at least both parents (n=67). Similar calculations were made for all mutations in *LRRK2* combined, on the basis of results for 1387 patients in 152 families and 94 singletons.

**Figure 3:**
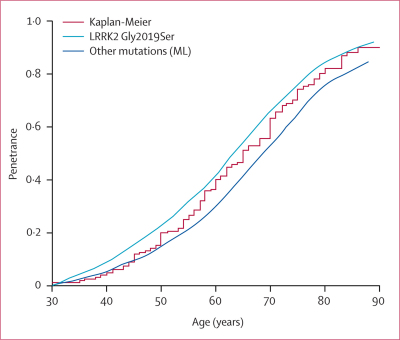
Age-specific risk of PD Risk is estimated with the Kaplan-Meier method for the whole sample and with the maximum-likelihood estimation (ML) for all patients with mutations in *LRRK2* combined.

Survival analysis was done on a subset of carriers after exclusion of all index cases (152). Kaplan-Meier analysis estimated the cumulative risk of PD as 36% at 59 years, 59% at 69 years, and 80% at 79 years. In the maximum-likelihood estimation, there were no significant differences in displacement by sex or ethnic group; however, there were significant displacements for patients with LRRK2 Gly2019Ser (mean displacement 1·66, SE 0·06; p< 0·02) and patients with the other mutations in *LRRK2* combined (mean displacement 2·00, SE 0·13; p<0·01). When this difference was taken into account, the cumulative risk for carriers of LRRK2 Gly2019Ser was 28% at 59 years, 51% at 69 years, and 74% at 79 years; the cumulative risk for carriers of LRRK2 non-Gly2019Ser mutations combined was 40% at 59 years, 64% at 69 years, and 84% at 79 years. Figure 3 shows the penetrance estimates calculated with the Kaplan-Meier and the maximum-likelihood methods.

## Discussion

Our prevalence data for *LRRK2*-associated PD imply that most clinicians who treat movement disorders will treat patients with this disorder at a clinically relevant frequency. For example, table 1 shows that 1% of white patients with sporadic PD and 3% with hereditary PD have the LRRK2 Gly2019Ser mutation, which means there is a prevalence of 3 cases per 100 000 people (estimate based on a PD prevalence of 200 cases per 100 000, and 15% of cases of PD are hereditary).^19^ This prevalence is similar to those for other neurological disorders, such as multiple system atrophy (4 per 100 000), progressive supranuclear palsy (6 per 100 000), motor neuron disease (6 per 100 000), and common single-gene disorders, such as Huntington's disease (2 per 100 000) and haemophilia A (5 per 100 000).^20^, ^21^, ^22^, ^23^ Moreover, 3 cases per 100 000 is an underestimate of the overall prevalence of mutations in *LRRK2* because it does not include mutations other than LRRK2 Gly2019Ser or the higher frequency of LRRK2 Gly2019Ser in particular populations (table 1).

The core feature of the 356 patients with LRRK2 Gly2019Ser-associated PD was asymmetrical, tremor-predominant parkinsonism with bradykinesia and rigidity that responded to dopamine replacement and functional neurosurgery. With respect to these symptoms, patients with LRRK2 Gly2019Ser-associated PD are indistinguishable from patients with idiopathic PD. Tremor was more common in patients with LRRK2 Gly2019Ser-associated PD, and abduction–adduction leg tremor should be regarded as a useful diagnostic clue.

Non-motor symptoms generally occurred at similar frequencies in patients with LRRK2 Gly2019Ser to those reported for patients with idiopathic PD; however, patients with LRRK2 Gly2019Ser had a lower risk of cognitive impairment and hyposmia than did patients with idiopathic PD. The patients who had normal olfaction are an interesting subgroup that might have had their olfactory pathways spared. Owing to insufficient reliable olfactory data in the QSBB series, we were unable to make comparisons; however, to detect anosmia in only 51% of patients with mutations in *LRRK2* is unexpectedly low when olfactory dysfunction is reported in 80–100% of patients with idiopathic PD.^8^, ^24^

Several factors imply that LRRK2 Gly2019Ser-associated PD is less severe than idiopathic PD. A more benign progression was seen: 6% of patients at Hoehn and Yahr scale stage 1 and 31% of patients at stage 2 had had PD for longer than 10 years. Another factor is a longer time to the first fall. Furthermore, patients with idiopathic PD needed dopamine-replacement treatment earlier than patients with LRRK2 Gly2019Ser did and were more prone to drug-induced dyskinesia.

Patients with LRRK2 Gly2019Ser-associated PD had a greater propensity to dystonia than did patients with idiopathic PD. Although this dystonia was nearly three times more probable in the first 2 years of the disease, it was seen only rarely in patients with LRRK2 Gly2019Ser-associated PD before dopamine-replacement treatment. This distinguishes LRRK2 Gly2019Ser-associated PD from PD due to recessive genes, in which pretreatment dystonia can be prominent.^25^, ^26^, ^27^

LRRK2 Gly2019Ser-associated PD can be easily distinguished from *PARK2*-associated PD on the basis of an older age of onset; the symptoms of PD were rare before 40 years in patients with LRRK2 Gly2019Ser-associated PD, whereas symptom onset after 40 years was rare in patients who were homozygous for mutations in *PARK2*. This rule of thumb has clinical uses, particularly in the genetic counselling of patients with sporadic PD and their affected siblings, in whom the mode of inheritance is often unclear.

A limitation of this study is that despite careful efforts to standardise data collection, the data are derived from international tertiary centres rather than from one community-based population, which introduces the possibility of bias.^28^

Until now, six mutations in *LRRK2* satisfy the requirements for proven pathogenicity as defined by the consortium. Until new data are reported, clinicians should be cautious about any interpretation and advice they give to patients with regard to other mutations in *LRRK2*, particularly because many of the non-synonymous mutations that were originally reported as pathogenic have been proven not to be. The webtable lists the consortium members who offer diagnostic (non-research) testing of *LRRK2.* A list of alternative testing sites is also available. Because exon 31 (Arg1441Cys, Arg1441Gly, Arg1441His) and exon 41 (Gly2019Ser, Ile2020Thr) are mutational hotspots, many institutions restrict testing to these exons.

Previous estimates of *LRRK2* penetrance were based on smaller sample sizes than reported here, and whether probands were included is not clear in some studies.^5^, ^7^, ^29^, ^30^, ^31^ The selection bias was minimised in this study by excluding the proband for all families and only including the families of sporadic (singleton) cases if there were data available on at least both parents. The authors of previous studies reported that the cumulative risk of PD by the age of 59 years ranges from 13–45%,^29^, ^31^ and from 21–85% at 69 years.^30^, ^31^ The range was widest at 79 years, with estimates from 32%^32^ to 100%.^5^, ^31^ Figure 3 shows the intermediate values from the consortium data; for example, a person who inherits LRRK2 Gly2019Ser has a 28% risk of PD at 59 years, 51% at 69 years, and 74% at 79 years. This reduced penetrance explains the high prevalence of mutations in patients with sporadic PD and the occasional occurrence of mutations in controls. There was no difference in penetrance by sex or ethnic group; however, there was a suggestion that carriers of LRRK2 Gly2019Ser had lower penetrance than did carriers of other *LRRK2* mutations. Because the carriers of the other mutations are under represented in our sample, more investigations need to be done to assess the specific effects of these mutations.

The high concordance between the Kaplan-Meier and the maximum-likelihood estimates corroborate the reliability of these results and highlight the need for the careful selection of patients to obtain unbiased estimates with the Kaplan-Meier method. Indeed, the inclusion of putative carriers who were not validated by the segregation analysis, sporadic cases without family data, or index cases in the survival analysis resulted in, on average, a 10% increase in the penetrance estimates at any age range.

Hospital-based and volunteer patient proband series have been criticised for overestimating mutation penetrance because they can over-represent multiplex families (families with many affected individuals), and the data from these might not be appropriate to use when counselling patients with sporadic PD.^33^ Multiplex families are also likely to share the combined influences of susceptibility genes and epigenetic and environmental factors, which increase risk but were not taken into account in the ascertainment adjustment. In this regard, our estimates are most appropriately used for the estimation of the age-specific cumulative risks of PD in patients with a known family history, either in those who have a mutation or in those who are at risk of having a mutation. Although the provision of penetrance data to the relatives of patients with sporadic PD who are carriers of mutations in *LRRK2* might be a challenge, if these relatives also carry the mutation, their risk of developing PD is at least no greater than the estimates in figure 3, and probably lower.

In general, we only recommend testing for the common LRRK2 Gly2019Ser mutation and only within the framework of pretest and post-test counselling. Without reliable penetrance estimates, we recommend that testing for other pathologically proven mutations is considered on a case-by-case basis.

There are two additional caveats. First, patients should be counselled that the frequency of mutations is greatly determined by ethnic group; for example, we showed that the common LRRK2 Gly2019Ser mutation is found in 10% of Ashkenazi Jews with sporadic PD and in 4% of Portuguese patients with sporadic PD but in only 1% of white North Americans. This means that it is probably only appropriate to test patients with sporadic PD who are in high-risk groups. Second, patients should be aware that the main benefit of testing is to improve diagnostic accuracy. The presence of a pathogenic mutation does not influence treatment choices, a point that also applies to the presymptomatic testing of individuals with a family history of PD, at least until such time as neuroprotective drugs are discovered.

## Web Extra Material

Webtable *LRRK2* consortium sites that provide diagnostic (non-research) testing of *LRRK2*
