# Supplementary material for: Phenotype, genotype, and worldwide genetic penetrance of LRRK2-associated Parkinson's disease: a case-control study
Source: Lancet Neurol. 2008 Jul;7(7):583–90. doi: 10.1016/S1474-4422(08)70117-0 (PMC2832754; doi:10.1016/S1474-4422(08)70117-0)
Supplement: Webtable — LRRK2 consortium sites that provide diagnostic (non-research) testing of LRRK2 [file mmc1.pdf]

Phenotype, genotype, and worldwide genetic penetrance of *LRRK2*-associated Parkinson's disease: a case-control study

Correspondence to:
Daniel G Healy, Department of Clinical Neurosciences, Institute of Neurology, University College London, London WC1N 3BG, UK
danhealy@doctors.org.uk

	Screening for mutations that cause
Department of Neurodegenerative Diseases, Hertie-Institut for Clinical Brain Research, University of Tuebingen, Tuebingen, Germany (thomas.gasser@uni-tuebingen.de)	Screen for specific mutations and complete *LRRK2*
UF de Neurogénétique, Departement de Génétique et Cytogénétique, Hôpital de la Salpêtrière, Paris, France (Cecile.Cazeneuve@psl.aphp.fr)	Only Gly2019Ser
Department of Neurogenetics, Institute of Neurology, Queen Square, London, UK (m.davis@uclh.nhs.uk)	Only Gly2019Ser
Departments of Human Genetics and Neurology, University of Luebeck, Luebeck, Germany (christine.klein@neuro.uni-luebeck.de)	Gly2019Ser and other proven pathogenic mutations if negative
Medical Genetics Laboratory, Foundation IRCCS, Ospedale Maggiore Policlinico, Mangiagalli e Regina Elena, Milan, Italy (labgen@policlinico.mi.it)	Gly2019Ser, Ile2020Thr, Arg1441Cys, Arg1441Gly, Arg1441His
Department of Clinical Genetics, Erasmus MC, Rotterdam, Netherlands (DNADIAGNOSTIEK.CL15@erasmusmc.nl)	Screen for specific mutations and complete *LRRK2*
Laboratory of Neurogenetics, Department of Neuroscience, Mayo Clinic, Jacksonville (farrer.matthew@mayo.edu)	Gly2019Ser, Ile2020Thr, Arg1441Cys, Arg1441Gly, Arg1441His, Tyr1699Cys

A list of other, non-consortium test sites is available on http://www.geneclinics.org

Webtable: *LRRK2* consortium sites that provide diagnostic (non-research) testing of *LRRK2*
